# External Factors Impacting Residents’ Participation in Waste Sorting Using NCA and fsQCA Methods on Pilot Cities in China

**DOI:** 10.3390/ijerph20054080

**Published:** 2023-02-24

**Authors:** Baihui Jin, Wei Li

**Affiliations:** School of Economics and Management, Taiyuan University of Technology, Taiyuan 030024, China

**Keywords:** household waste management, influencing factors, fuzzy-set qualitative comparative analysis, necessary condition analysis, residents’ participation behavior

## Abstract

Recycling waste is important as it can help to reduce environmental pollution caused by “waste siege”. Source classification is an important part of the municipal solid waste (MSW) sorting process. The factors that prompt residents to participate in waste sorting have been debated by scholars in recent years; however, there are not many papers that focus on the complex relationships between them. This study reviewed the literature that concerns residents’ participation in waste sorting, and it summarized the external factors that might influence residents’ participation. Then, we focused on 25 pilot cities in China, and we analyzed the configuration impact of external factors on residents’ participation using a necessary condition analysis (NCA) and a fuzzy-set qualitative comparative analysis (fsQCA). We found no consistency between variables, nor was there one single condition that caused residents to participate in waste sorting. There are two main methods (environment-driven and resource-driven) that can help achieve a high participation rate, and three methods that can cause a low participation rate. This study provides suggestions for the implementation of waste sorting in other cities in China, as well as developing countries, with an emphasis on the importance of public participation.

## 1. Introduction

Rapid industrialization and urbanization have not only led to economic growth and development, but they have also introduced many social and environmental problems, including growing amounts of municipal solid waste (MSW). In 2021, China produced approximately 248 million tons of MSW, which is 1.5 times the amount that was produced in 2004 (155 million tons); by 2030, 480 million tons of MSW are expected to be produced [1]. During the 13th Five-Year Plan period, the annual growth rate of domestic waste in China is expected to be 6%, and two-thirds of the Chinese cities that are experiencing rapid economic development are expecting to face the problem of “waste siege” [2]. Sorting waste from the source is considered to be an effective measure that can solve this problem; however, residents’ participation plays a significant role in this process.

MSW disposal generates significant greenhouse gas emissions (GHG); furthermore, it may be considered a waste of resources. In China, after the “Implementation Plan for Domestic Garbage Classification Systems” was introduced in 2017, MSW was divided into the following categories: wet waste (food waste), recyclable waste, hazardous waste, and remaining waste (dry waste). In accordance with the treatment strategy detailed in the plan, kitchen waste is treated using anaerobic digestion or composting methods; recyclable waste is treated using methods that will transform it into usable resources; and the remaining waste will be disposed of via incineration [3]. To promote sustainable urban development further, waste-to-energy (WtE) treatments, such as waste-to-liquid, waste-to-solid, and waste-to-gas fuels, are needed [4]; however, unsorted waste is slowing down the development of these treatments. Compared with other countries, China’s kitchen waste comprises a higher level of unclassified and water-rich substances [5]. The low calorific value and high moisture content of waste restricts its successful transformation into effective resources. China, which has set a goal of becoming carbon neutral by 2060, is under a great deal of pressure to reduce emissions; ensuring that waste is classified when it is generated will be an effective measure that can help reduce GHG.

Even though developed countries have provided us with a framework that demonstrates how to sort MSW, the act of consciously sorting waste is not habitual for most residents. Ensuring that recycling occurs in developing countries still relies on the use of mandatory policies [6]. Concerning waste management, China began to issue regulations in the 1990s. At the time, due to the enforcement of different regulations across the country, and a lack of infrastructure in certain regions, sorting waste did not gain much popularity [7]. In June 2016, the first plan for a compulsory waste classification system was implemented. In June 2019, a new regulation was implemented, which established waste classification and treatment systems in 46 pilot cities above the prefecture-level. As a result, Shanghai, a pioneering city in terms of its response to the new directive, witnessed a rapid increase in the sorting rate [8]. Other pilot cities have gradually introduced local regulations; however, there are still difficulties regarding residents’ participation behavior.

At present, pilot cities are focusing more on the back-end processing of MSW, rather than concentrating on reducing consumption and recycling, which ought to be the main goal of a developing circular economy. In accordance with previous research, more than 50 percent of people in China are willing to support the government’s recycling program; however, waste sorting remains inefficient due to a lack of information on waste classification [9]. Another reason for residents’ non-participation in the recycling program may be the establishment of inflexible policies that fail to take the different levels of economic development and population development in different regions into account [10]. In Chinese cities, 3.3–5.6 million people (0.56–0.93% of the urban population) produce waste as a result of their participation in the informal sector [11]. The impact of the informal sector on the environment is twofold. On the one hand, the informal sector can reduce the cost of waste disposal and create more jobs. On the other hand, the non-standard methods of waste disposal used by this sector may cause the further deterioration of the environment; in turn, this may increase the likelihood of developing common health problems by 1.7 times [12]. Therefore, in order to change the current situation and improve residents’ participation in recycling programs, it is crucial to understand the factors influencing their non-participation.

Many researchers have explored the factors influencing residents’ participation in the waste sorting process. A person’s participation in waste sorting, recycling, waste collection, and waste treatment processes is most directly affected by their intention to participate in these processes [13]. Various studies have illustrated the internal factors that prompt people to move from intention to behavior; these include subjective norms, a person’s attitude, a person’s sense of belonging, and the perceived convenience of performing a certain action, among others [14,15]. Some scholars have found that the influence of external factors on a person’s intrinsic motivations further stimulate the likelihood that they will act on their intentions to implement pro-environmental behaviors [16].

Moreover, the likelihood of a person engaging in behaviors that assist with the waste sorting process is determined by their external environment, which is created independently of the individual. The external factors exerting pressure on people’s behaviors mainly include social governance, economic incentives, social norms, infrastructure convenience, and so on. Occasionally, scholars reach different conclusions about the individual effects of these factors. Areas that have rapidly developing regional economies, for instance, tourism hubs, produce more waste, which is not conducive to the waste sorting process [17]. Furthermore, higher taxes always means more investment in infrastructure. Infrastructure has a positive, moderating effect on the relationship between willingness and behavior [18]. Different aspects of infrastructure, such as design preferences, physical designs, and visual prompts, also have a significant effect on public participation [19]. Moreover, urban policies play a crucial role in whether people decide to participate in the waste sorting process; indeed, differences in the population, the unemployment rate, and geographical factors cannot be ignored [17]. Some studies have shown that people living in areas with waste sorting policies showed higher levels of participation in waste sorting activities than people living in areas without waste sorting policies; furthermore, knowledge of the waste sorting process, and a willingness to participate, have a mediating effect on the participation levels [10]. Policies offering financial incentives are very effective; however, regarding such policies, participation levels are influenced by the interpersonal relationships in a community. For example, in communities with a strong, volunteering ethos, financial incentives can negatively affect recycling performance [20]. Legislation and education can also affect the participation rate by improving the values and beliefs in a population; for instance, it may impact the level of responsibility that people may feel toward their own waste production habits. Nevertheless, legislation and education can also have different spillover effects on the participation rate [21]. To sum up, the factors influencing public participation are not always consistent.

Regarding the abovementioned influencing factors, scholars have tended to study the one-way linear effect of residents’ participation behavior; few previous studies have focused on the interactions between these factors. Indeed, Khalil et al. examined the interaction between digital precision and the manner in which information is framed and the impact it had on residents’ awareness of food waste [22]. Chen et al. explored the interaction between information and the perceived convenience of performing a certain action and the impact it had on waste separation behavior [23]. Most research concerning methods of adequacy and necessity tend to be focused on enterprise management and decision-making [24]; moreover, such research is also used to help make policy decisions, among other things [25]. In this paper, a combination of qualitative and quantitative methods are used to study the interactions between external factors affecting the residents’ participation in waste sorting; these methods include the necessary condition analysis (NCA) and the fuzzy-set qualitative comparative analysis (fsQCA). There are strong reasons for choosing methods such as the NCA and fsQCA. The NCA method was applied in order to measure sustainable behavior [26], the mediating effects on participation levels [27], and so on. Furthermore, prior to this study, the NCA had not been applied to studies focusing on waste classification behavior. We believe that this method provides a novel theoretical and statistical basis for analysis. The FsQCA method has been widely used in business management [28] and information management [29]. It is suitable for both multiple attribute variable investigations and specific case details [30]. China’s territory is vast, and the development rate of its waste management infrastructure is not the same in different regions. Combining the two methods ensures the completeness and robustness of the results. It is helpful to understand the complex relationships between the different external factors affecting residents’ behavior with regard to waste sorting.

Some achievements have been made in studies that focus on MSW and recycling behavior. However, there are still some shortcomings, which are as follows: (1) previous research has investigated the factors directly influencing waste classification and recycling behaviors; however, such investigations were one dimensional in that they failed to account for multiple external environmental factors and the effect that they have on residents’ participation in waste sorting and recycling; and (2) most studies failed to consider the interactions between external factors and their effects on residents’ waste classification and recycling behaviors. The research questions of this study are as follows: What are the external factors that affect residents’ waste classification behavior? How do these factors interact with one another? Based on these problems, a new model to assess the factors influencing residents’ waste classification and recycling behaviors was constructed in order to explore how to influence residents’ waste sorting behavior in the future. 

More specifically, we focused on the issue of resident participation in sorting waste, noting whether it was sorted as it was generated. The pilot cities have different resources and conditions, and the governing bodies of each city have also chosen different policies with which to implement proper waste management; therefore, this study aims to assess the current waste classification practices of residents and whether they participate in those practices. Additionally, with a focus on the pilot cities in China that have these waste classification systems, this study identifies the interactions between the key influencing factors. Most studies examining these influencing factors have focused on the single linear effects of residents’ participation in the MSW. Few studies have studied the sufficiency and necessity of these factors, nor the interactions between them; therefore, this study provides empirical examples for governments in regions with different circumstances in order to promote further public participation in waste sorting processes. The following sections are structured as follows: section two details the manner in which our hypotheses were developed; section three introduces the methods through which we collected data and conducted analyses; section four describes the results of our data analyses, and furthermore, the results are verified; the results, future implications, and prospects for future research are discussed in section five; and finally, section six details our conclusion.

## 2. Hypotheses Development

### 2.1. Economic Development

There is a convergence between the growth of waste generation and GDP growth [31]. Economic growth creates a lot of waste, especially in high-income and upper-middle-income countries [32], which exacerbates environmental problems [33]. Moreover, the ontological insecurities of those in low-income economic brackets indicate that they feel as though they are not in control, which subsequently leads to excessive amounts of waste being produced by such consumers [34]; however, Liu et al. found that a synergistic consideration of the GDP growth rate, an urban construction policy, and a tourism development policy can significantly reduce increases in MSW [35]. In developed regions, people are more likely to abide by environmental commitments made by the government and corporations than in poor or developed regions [36]. In addition, the level of GDP measures the degree of national wealth, and regions with a high GDP have more tax revenue with which to build recycling facilities. Economic development is considered to be an important condition affecting residents’ participation in waste sorting processes [37]; thus, we propose our first hypothesis:

**H1:**  *Economic development is a necessary external condition for ensuring residents participate in waste classification processes*.

### 2.2. MSW Policies

Policy is a top-down approach that directly constrains and motivates classification behavior. Under the guidance of central policies, all regions formulate additional policies that are in line with local development. The main types of waste classification policy are mandatory policies and incentive policies. Laws and regulations impose fines on violations; fines are mainly imposed on residents who exceed garbage discharge standards or fail to classify waste properly. Moreover, these additional fees (such as weight fees) directly regulate people’s behavior [38]. Incentive policies usually cooperate with the points exchange system and the unit pricing system in order to reward residents who participate in the waste classification scheme [38,39].

Policies can either affect behavior directly (as external factors) or they can affect a person’s internal, individual decisions (as subjective norms) [10]. Residents’ recognition of the effectiveness of government’s waste classification policies can effectively increase their willingness to participate in the waste classification process [40]. Publicity and education can enrich individuals’ knowledge on this subject, which thus indirectly influences their behavior [10].

In accordance with the characteristics of MSW, pilot cities have formulated their own measures for waste classification and management. When considering the waste management models in China, we divided policies into two groups: mandatory policies and advocacy policies (details in Section 3.2). Compulsory policies are more effective than advocacy policies in terms of the standard rate at which MSW is classified, and the standard rate at which MSW resources are utilized [41]; however, in the early stages of the implementation of mandatory policies, residents may experience negative emotions [42]. Although some studies suggest that economic incentives are more effective than social mobilization policies (such as education policies), other studies have shown that the effectiveness of economic incentives is also related to other factors [43]; for example, the effect of cash rewards on low-income families is perhaps more obvious than it is on high-income families [44]. We propose two sets of hypotheses concerning mandatory and advocacy policies:

**H2:**  *Mandatory policies are a necessary external condition for ensuring residents participate in waste classification*.

**H3:**  *Advocacy policies are a necessary external condition for ensuring residents participate in waste classification*.

### 2.3. Environmental Quality

Intending to enact positive environmental change strengthens waste recycling practices [45]. Several studies use the Extended Theory of Reasoned Action (TRA) to explain pro-environmental behavior; indeed, concern for the environment positively affects waste sorting behavior via an intermediary variable (guilt) [46]. Environmental concern indicates that a person feels strongly about protecting the environment [47]. People tend to recycle when pollution, climate change, and resource depletion affects the wellbeing of individuals or groups [48]. Environmental concerns comprise a person’s attitude towards the environment [49]. The Theory of Planned Behavior (TPB) has been widely used in the field of environmental behavior research when investigating pro-environmental behaviors and environmental concerns [50]. Xu et al. expands on the TPB to show that positive environmental attitudes can enhance pro-environmental behaviors [51]. In addition, residents’ psychological perceptions of MSW pollution also significantly improved their willingness to participate in the waste sorting process and the degree to which they classified waste [52]. By measuring environmental quality, to some extent, we were also able to measure the capacity and success of waste treatment; thus, we propose the following hypothesis:

**H4:**  *Concern for environmental quality is a necessary external condition for ensuring residents participate in waste classification*.

### 2.4. Convenience

Perceived behavioral impairments have been shown to have a significant impact on environmental behaviors, such as the time, physical cost, and convenience of performing an action [53]. Convenience refers to the time taken to perform an action, how comfortable performing that action is, and how simple it is to perform that action [54]. The higher the perceived level of convenience, the more likely it is that residents will adopt waste classification habits, because of the fewer required behavioral changes, and the less time it takes for the group to reach a stable decision-making state [23]. Regarding the management of sorting waste as it is generated, many measures have been applied to increase residents’ participation in this process. In Sweden, food waste collection hangers have been installed in each household [55]; in California, consumers prefer nearby recycling centers with flexible hours and short waiting times [56]; and in south Korea, a reverse vending machine (RVM) was proposed for use in convenience stores [57]. Therefore, this study argues that the perceived convenience of recycling has an important influence on residents’ participation in the waste sorting process.

**H5:**  *Convenience is a necessary external condition for ensuring residents’ participation in waste classification*.

## 3. Data and Methodology

### 3.1. Literature Research

**Figure 1:**
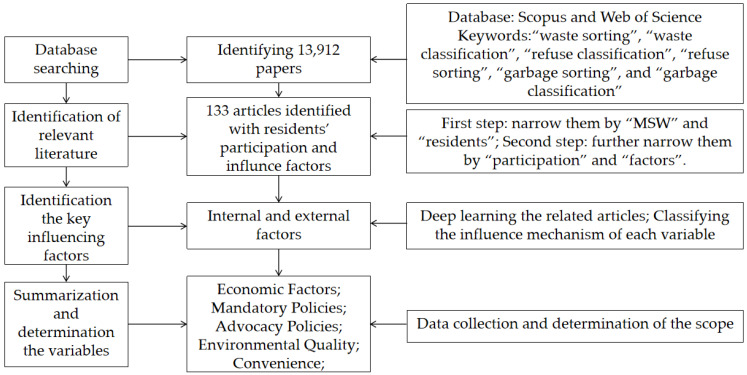
The literature research process for identifying variables.

Firstly, data concerning waste classification and resident participation were collected from the literature. Additional information from the Scopus database and Web of Science database was used to support the findings in the literature. We searched for the following keywords: “waste sorting”, “waste classification”, “refuse classification”, “refuse sorting”, “garbage sorting”, and “garbage classification”. Overall, 13,912 articles qualified for use in this study; then, we narrowed the number of articles down. The terms “municipal solid waste and resident” were used to narrow down the search results, and as a result, 259 articles were selected. Next, the terms “participate” and “factor” were used to further narrow down the results, and the final 133 results were scanned in depth to ascertain the key factors driving residents’ participation in waste classification (supplementary materials). Figure 1 shows the literature research process. Regarding the observed factors, we classified and summarized them, and this provided a basis for our subsequent research. Finally, the variables we adopted are described in detail in Section 3.2.

### 3.2. Measurement

(1) Economic Factors

When ascertaining the economic factors affecting residents’ participation in the waste classification process, we selected GDP per capita as the economic factor that would measure the economic development of a region.

(2) Mandatory Policies and Advocacy Policies

In order to determine which factors could influence the participation of residents in waste classification processes in the pilot cities, we reviewed municipal policy documents. We visited the websites of the governments of local municipalities in order to browse and download policy documents on waste classification, including laws, regulations, announcements, and measures, among others. After reading each document in-depth, the categorization standards were clearly defined and are shown in this paper. We defined mandatory policies as those that ensure “compulsory participation in waste classification processes”, or as those that burden people with legal liabilities if they refuse to implement waste sorting behaviors. For instance, an example of a Taiyuan municipal household waste management regulation is as follows: if the unit “fail[s] to put household waste into the designated collection points or collection containers, the unit shall be fined…no less than 5000 yuan and no more than 50,000 yuan; Impose a fine of no less than 50 yuan [and] no more than 200 yuan on individuals” [58]. If policies contain the words “encourage,” “guide,” or “educate”, among others, and if policies reward categorized residents, then they are considered to be advocacy policies. For example, an instance of an advocacy measure in Shenyang is as follows: “to establish a green integra[ted] system” [58]. The number of policies issued by each local city before 2020 is displayed in the Appendix A.

(3) Environmental Quality

Environmental quality, as a driving force behind municipal waste treatment, was selected for assessment in this study [59]. We calculated the environmental quality using the weighted linear method, as referred to in [60]. The detailed process used to calculate the environmental quality involves the following two steps:

The first step requires the calculation of the adjustment coefficient, A*, which is as follows:

(1) $$A_{ij}=\frac{P_{ij}}{\sum_{i}P_{ij}}/\frac{PGDP_{i}}{\sum_{i}PGDP_{i}}$$

(2) $$A_{ij}^{*}=A_{ij}/\sum_{i}A_{ij}$$

where *i* indicates the number of pilot cities. *j* = 1, 2, and 3 and represents the ratio of three types of treated pollutants, including a comprehensive utilization rate of industrial solid waste, a centralized treatment rate of sewage plant, and a harmless treatment rate of domestic waste, respectively, as follows: the comprehensive utilization rate of industrial solid waste, the centralized treatment rate of sewage, and the harmless treatment rate of domestic waste. *PGDP_i_* is the GDP per capita of each city. *P_ij_* refers to the treatment rate of pollutant *j* in city *i*. Then, in accordance with the linear weighted method, the environmental quality (*EQ*) is calculated as

(3) $$EQ_{i}=\sum_{j}A_{ij}^{*}P_{ij}$$

(4) Convenience

Waste classification facilities provide the basis for residents’ sorting and recycling activities. These factors further affect people’s participation in waste classification processes at the psychological level, as an external factor [61]. Limited by the availability of data, we used waste collection vehicles to measure the convenience of urban sorting facilities.

### 3.3. Data Sources and the Research Area

The data we used comes from China’s urban statistical yearbook, the statistical yearbook of Chinese urban architecture, and provincial statistical yearbooks. All the data were reviewed, and most of the missing data were supplemented by provincial and municipal statistical yearbooks. The few missing values were supplemented using interpolation methods. 

**Figure 2:**
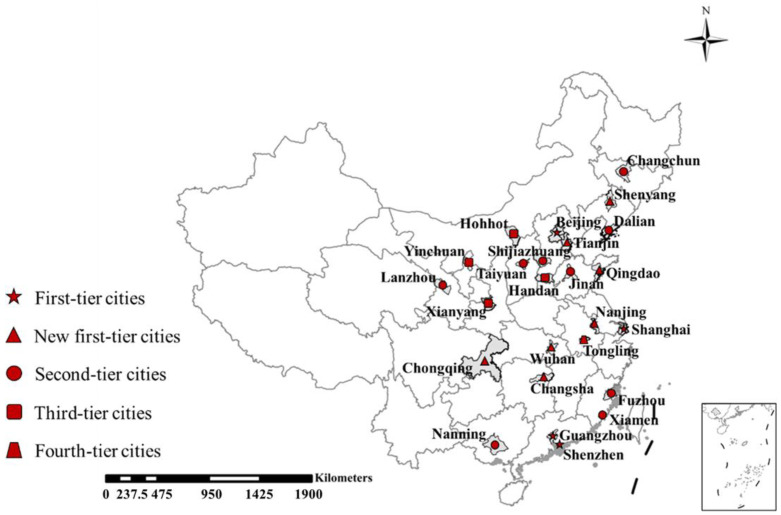
The geographical locations of the waste classification pilot cities.

The data concerning residents’ participation in waste classification were derived from previous studies in the literature, local government website reports, municipal congress government website reports, the news, and newspapers. Based on data availability, 25 of the pilot cities were finally selected: Beijing, Tianjin, Shijiazhuang, Handan, Taiyuan, Hohhot, Shenyang, Dalian, Changchun, Shanghai, Nanjing, Tongling, Jinan, Fuzhou, Xiamen, Qingdao, Wuhan, Changsha, Guangzhou, Shenzhen, Nanning, Chongqing, Xianyang, Lanzhou, and Yinchuan. Figure 2 shows the geographical locations and classification rates of the selected waste sorting pilot cites. These cities cover most provinces, and, to some extent, they may represent the waste classification situation in other areas. 

### 3.4. Necessary Condition Analysis (NCA)

Necessary condition analysis (NCA) is an approach that aims to identify a single necessary condition. More specifically, unlike the regression approach where “A affects B”, the NCA approach is based on the principle that “without a certain level of A there is no B” [62]; therefore, any condition must affect the outcome. Moreover, the influence of a variable has to reach a certain level in order to be able to produce results. To assist with this analysis, we referred to Dul’s research [63]. 

The ceiling Y=f(x) was set in order to divide the area into two, so that one part represented an area with cases, and the other represented an area without cases. Y≤f(x) was the lower area representing the feasible cases that comprise the necessary conditions; exceptions up to 5% were permitted. Two techniques are used in NCA to draw the ceiling line: Ceiling Envelopment-Free Disposal Hull (CE-FDH) and Ceiling Regression-Free Disposal Hull (CE-FDH) [62]. Of these techniques, CR-FDH technology is applicable to both the continuous variable and the discrete variable; although, CE-FDH technology is suitable for binary variables and discrete variables. If the upper envelope line contains all of the observed values, the accuracy is 100%.

The bottleneck level refers to a certain level that indicates if the maximum observation range has been reached; therefore, the value level needs to be satisfied within the maximum observation range, as delineated by the antecedent conditions. The bottleneck level is subject to the ceiling line, and it can indicate the extent to which a condition can help achieve a desired result [64].

### 3.5. Fuzzy-Set Qualitative Comparative Analysis (fsQCA)

The fsQCA method is based on qualitative comparative analysis (QCA). It is a set analysis method that deconstructs the phenomenon of causal complexity, and it is used to determine the sufficient and necessary causal conditions under which an outcome may occur [65]. To identify the configuration paths, we took each city as a separate case, and we analyzed it using the fsQCA software package, ensemble analysis methods, and truth table techniques.

#### 3.5.1. fsQCA Calibration

Before calibrating the data, we translated the variables into sets. This was carried out in order to establish a threshold with which to evaluate the combination of conditions that impact the various outcomes.

A fuzzy membership degree of 1 means that the case completely belongs to the set (completely in the set), whereas a fuzzy membership degree of 0 means that the case completely does not belong to the set (completely outside the set). A fuzzy membership degree (also called the intersection point, intermediate point, or maximum fuzzy point) of 0.5 means that a case both belongs and does not belong to the fuzzy set. The threshold can be adjusted in accordance with the different characteristics of each case.

Each condition and outcome is regarded as a fuzzy set, and cases in these sets are given a corresponding membership score; this is the calibration process [66]. During the calibration process, a variable value of the case needs to be calibrated in accordance with the fuzzy-set membership degree (from 0 to 1) so that it can become a set [67]. Ideally, studies should be calibrated directly in accordance with external standards. With the indirect calibration method, it is necessary to rescale the measurement results in accordance with existing theories on the basis of qualitative evaluation [67]. As there is no clear theoretical or external knowledge providing guidance on this specific area of study, and considering that the cases in this paper had good national representation, calibration was carried out in accordance with the data of the case itself; therefore, this paper adopted the direct calibration method in order to convert the data into a fuzzy-set membership score.

To calibrate the data, we chose 0.95, 0.50, and 0.05 as the three thresholds, the data were converted into values between 0 and 1 [68], and the causal conditions and data pertaining to the original results were converted into fuzzy-set scores. In addition, in order to avoid situations wherein cases are difficult to classify, and thus, cannot be analyzed, the sets with fuzzy membership scores of 0.5 were adjusted by adding 0.01 to its membership score, if the score was less than 1 [69]. The results of the calibration anchor points for each variable are shown in Table 1.

#### 3.5.2. fsQCA Analysis

We used the truth table technique to analyze the different conditions affecting residents’ participation in waste sorting processes. We listed all the logical combinations of the selected variables to form the rows of the truth table, and we calculated the membership score of each row using fuzzy mathematics. Truth table plot recipes were used for each antecedent configuration. Using the truth table, we analyzed the impact of different configurations of external factors on residents and their participation in waste sorting. 

To evaluate the configurations of external factors, coverage and consistency are both important criteria. The key to performing this analysis is to find a balance between consistency and coverage. Coverage refers to the share of results that can be explained by each solution term versus the share of results that are explained by the solution in its entirety. For fuzzy sets, consistency is used to measure subsets of relationships; in other words, this refers to when the membership score in a given set of causal attributes is always less than, or equal to, that in the results set. Although coverage is used to measure correlation, it also reflects the proportion of consistent members out of the total number of members in the results set. Ensemble consistency is measured based on fuzzy membership scores; the formula is as follows:

(4) $$Consistenc\;y(X_{i}\leq Y_{i})=\sum \left(\min(X_{i},Y_{i}\right))/\sum \left(X_{i}\right)$$

where *i* represents the combination of conditions; $X_{i}$ represents the membership score of the combination of conditions; $Y_{i}$ represents the membership score of the outcomes; and min indicates the lower value between *X* and *Y.* Consistency assesses the membership scores of the combination of conditions and the set of outcome; higher scores indicate a better level of consistency. In general, the consistency of the conditions was greater than 0.75 [62].

We used the Proportional Reduction in Consistency (PRI) score to assess which configurations are consistent when fuzzy-set theory is applied, and which configurations are inconsistent. Configurations with a PRI score equal to, or exceeding, the critical value were considered to be fuzzy subsets of the outcome, and they were given a score of 1; otherwise, configurations were given a score of 0.

Program analysis can produce three kinds of solutions, which are complex solutions, parsimonious solutions, and intermediate solutions. Complex solutions do not use configurations that are logically feasible, and they are not often used in practical cases. Parsimonious solutions use all logically possible configurations without evaluating how rational it is to use these configurations. The intermediate solution uses logically possible configurations with empirical examples; it is considered superior to the other solutions [63] due to the lack of evidence and theories supporting the idea that environmental conditions affect the direction of the results. The results of the configuration analysis assume that the occurrence of a single external condition can influence, contribute to, and improve residents’ participation in waste sorting. The core conditions of each solution are identified by comparing the nested relations with the intermediate solution and the parsimonious solution. The conditions that appear in both the intermediate solution and the parsimonious solution are the core conditions of the solution, and the conditions that only appear in the intermediate solution are the edge conditions.

## 4. Results

This paper selects five conditional variables in order to explore what affects residents’ participation in waste classification. The variables and their descriptive statistics are shown in Table 2.

### 4.1. Necessary Condition Analysis

An NCA can identify whether an explanatory variable is a necessary condition impacting the results, and it can identify the overall impact of a particular result. Table 3 shows the results of the NCA analysis. Although the accuracy of the CR-FDH technology was not 100%, the overall impact was not significant. Due to the characteristics of the variables, this study opted to use CR-FDH technology for analysis purposes [63]. Overall, except for the convenience variable, the level of impact of the other variables was greater than 0.1; however, the *p*-value test for each variable was not significant, with values of 0.05, thus indicating that these variables were not necessary conditions that encouraged residents to participate in waste classification.

Next, the bottleneck report results were analyzed. The necessity analysis showed that no single factor was a necessary condition that contributed to improving residents’ participation in waste classification; therefore, the combined effect of each variable was further examined through a bottleneck analysis. The results are shown in Table 4. It shows the different variables increasing incrementally. In order to calculate a value for the participation rate, the combination of conditions comprising the bottleneck value should be met; for instance, no variable is required to achieve a membership score of 40%, with regard to the participation rate. To calculate the value representing the maximum degree of participation, it is necessary to take the environmental quality > 80.6%, economic factors > 88.0%, convenience > 82.1%, mandatory policies > 42.2%, and incentive polices > 46.8% into account.

Finally, we used the fsQCA method to verify the necessary conditions. We considered the consistency of a single variable to find the outcome variable. For fuzzy sets, the conditions and results have a partial membership relationship. The results concerning the participation rate and non-participation rate are shown in Table 5. No single variable at the consistency level passed the consistency threshold of 0.9 [70]; therefore, we may assume that there is no single condition affecting the participation rate and non-participation rate. The necessity and consistency of each individual variable and the combination of variables require further analysis.

### 4.2. The Configuration Analysis

Generally, a threshold of 0.80 is considered sufficient in terms of adequacy and consistency values [71]. In this paper, the original consistency threshold was set as 0.80, the PRI consistency threshold was set as 0.70, and the case frequency threshold was set as 1. The truth table is shown in the Appendix A. 

The results of the FsQCA analysis are shown in Table 6. There are four configuration pathways (H1–H4) pertaining to high participation rates, and of these, there are two main pathways. H1, H2, H3, and H4 constitute the second-order equivalent configurations, respectively, and their core conditions are the same. Their coverage score was 0.76 and their consistency score was 0.88. There are three configuration pathways pertaining to low participation rates: (1) low environmental quality together with low mandatory policies and low convenience, named NH1; (2) low environmental quality with low advocacy policies, named NH2; and (3) low convenience with high environmental quality, named NH3. The coverage score for these pathways was 0.691, and the consistency score was 0.89.

To ensure the stability of the results, we tested the robustness of the results. First, it must be noted that in removing the single necessary causal condition, the configuration remains unchanged. This step was not required because necessary causal conditions do not exist in this study. Secondly, robustness was tested by adjusting the consistency values. When we set the PRI consistency values to 0.8 and 0.85, respectively, the four pathways remained unchanged. As municipalities and first-tier cities have resource-based advantages and policies that strongly encourage participation in the waste sorting process, we deleted six cases from municipalities (Beijing, Shanghai, Tianjin, and Chongqing) and first-tier cities (Beijing, Shanghai, Guangzhou, and Shenzhen); despite this, the four pathways remained unchanged. Therefore, the configurations obtained using fsQCA analysis in this paper are reliable and robust.

## 5. Discussion

### 5.1. Discussion of Research Results

In this study, we assessed the impact of external factors on residents and their participation in waste sorting. In accordance with the NCA results, no single conditional variable constitutes the core factor prompting residents to participate, or not participate, in the waste sorting process; then, we used the fsQCA method to distinguish between the configuration paths. As per the results, there are two main combinations of factors that can achieve a high participation rate: high environmental quality together with few advocacy policies, and many advocacy policies together with high convenience. Moreover, we found that the main reason for the low participation rate is poor environmental quality. Other reasons include the inconvenience of MSW and policy problems. Overall, no single factor is sufficient or necessary to explain the participation rates concerning waste sorting.

The main pathway culminating in a high participation rate comprised the following combination of factors: high environmental quality and few advocacy policies. Both H1 and H2 comprise cases wherein cities exhibit a low economic performance, and less waste is produced as a result of household consumption. In these cities, the process of waste sorting is slow, and local government policies tend to be based on publicity and education. Advocacy policies concerning environmental quality influence residents’ participation in recycling processes; however, other policies could still be more effective. This result is similar to previous findings [72]. Based on the utility perception theory, a determinant of classification behavior is the perception that one may receive personal rewards, not only in terms of harvesting certain benefits, but also in terms of achieving a certain goal [73]. Good environmental quality will increase people’s confidence in waste sorting. Tongling is a typical example of a city following path H1. In the absence of too many mandatory policies, Tongling has a high participation rate; this is due to the fact that 1374 household waste classification centers have been built, and thus, the coverage rate concerning residential facilities reached 100%. H2 indicates that convenience may also be considered as an edge condition. Nanning and Lanzhou are the representative cities following this path. Since 2021, the allocation rate of household waste classification facilities in Nanning has reached 100%, with more than 1000 waste collection and transportation vehicles allocated for waste classification purposes, in accordance with the four categories of waste. Moreover, as per the responsibility of the sanitation station, if there is unclassified waste, the station can input the two-digit code that alerts the authorities who are tasked with fine management. 

The second main pathway culminating in high participation comprises the following combination of factors: high levels of convenience and a high number of advocacy policies. This conclusion is consistent with previous findings in the literature [74]. Incentive-based recycling policies have a ‘crowding out’ effect, and this can negatively impact other waste policies by reducing public support for other waste policies; however, the prescience of advocacy policies will disappear as other policies expire [75]. Moreover, H3 and H4 show different results when subjected to other conditions. This suggests that a high number of compulsory policies is not necessary; this finding differs with some studies [41]. H3 is always suitable for large cities that have a robust economy together with adequate infrastructure and a good waste management system. Cities that follow H3 include Beijing, Shanghai, and Qingdao. In 2015, Qingdao generated 2.21 million tons of municipal solid waste, which increased to 3.1 million tons in 2020, and it will increase to 6.5 million tons in 2035 [76]. The average efficiency of waste collection in these cities, in accordance with the standards of the four categories, was 75.7% [77]. An efficient recycling system reduces the cost of recycling. H4 illustrates that incentive-based policies, including smart device investments and returns in the form of points or cash, play a significant role in promoting participation in waste sorting. Typical cities that follow H4 include Changchun. Changchun has established a waste collection network comprising mobile trucks, which is an informal recycling model that recycles 95–96% of the waste [78]. This mode of waste collection integrates individual and household recycling; it not only improves residents’ enthusiasm toward waste sorting, but it also provides economic benefits for these individuals. This waste collection process is conducive to forming habits and routines relating to recycling waste. 

We also examined the combination of urban conditions that produce low participation rates. NH1 and NH2 represent low environmental quality, and the combination of factors that produce low participation rates are as follows: few mandatory policies and low levels of convenience. This shows that without a waste recovery system, neither a nice living environment nor strong policy guidance can prompt residents to spontaneously separate their waste. NH3 shows that poor environmental quality and back-end waste disposal leads to residents having less confidence in the waste classification result. This is in line with existing research focusing on developing countries, such as the Philippines, despite efforts by cities to implement effective measures that comply with legal regulations. Failures of waste management in this country are due to the lack of disposal facilities, including a lack of adequate soil cover and sanitary landfills for non-biodegradable materials [79].

### 5.2. Future Implications

From the results, the effect of a single causal condition may be positive or negative, depending on how it is combined with other conditions. Based on the above results, this study proposes the following policy recommendations. In order to increase the participation of residents in waste sorting, the combination of measures (such as those pertaining to environmental quality, infrastructure, and economic level) needs to be differentiated and flexible to match local objective conditions; for example, in cities with rapid economic development, such as Beijing, Shanghai, and Qingdao, more convenient facilities should be built to minimize the time spent depositing waste, and more sorting points should be set up to minimize the time spent and the economic costs incurred by residents. Furthermore, launching sorting points to exchange equipment and bestow economic rewards or honors on enterprises and individuals who demonstrate excellent levels of waste sorting would be an effective measure. 

For cities with low levels of economic development, such as Lanzhou, improving residents’ happiness is a better way to effectively improve environmental quality. As citizens become increasingly aware of the relationship between the environment and their own health, as a bottom-up approach, public participation cultivates the residents’ sense of identity and responsibility [80]; however, groups belonging to a lower socioeconomic bracket tend to spend less time on recycling activities because they have more pressing needs. Therefore, their sensitivity to policies is weak. On the other hand, financial incentives can be effective drivers for low-income households as they might benefit from the sale of recyclable materials. Positive fiscal incentives are easy to accept, but negative incentives (such as taxes and fees) are counterproductive. In addition, although these municipalities have implemented mandatory sorting policies and even fines, the entities that must abide by such measures are collectives and organized waste producers. In fact, individual residents are not subject to punishment. At the community level, due to the lack of effective implementation and supervision, despite any previous potential effort, waste is mixed once again, and residents lose their enthusiasm and confidence in the sorting process.

A high number of compulsory policies does not necessarily achieve a high participation rate. In accordance with our results, GDP levels are usually accompanied by compulsory government policies. Cities with high GDPs usually mean better infrastructure and more experience and leadership with regard to waste recycling; therefore, governments often adopt strong enforcement policies. However, in the long-term, it is difficult to maintain a high participation rate with only strong policies. Policies emphasizing punishment should be used with caution because of the risk of negative spillover effects [81]. The government should devise waste sorting policies in accordance with the characteristics of different regions, delivering waste classification education in schools, and raising awareness of waste sorting in the community, among other activities.

In accordance with the results of the existing research, an important driver behind consumers’ waste classification behavior is environmental concern (such as satisfaction with environmental quality, based on the “broken window theory”), and the most important barriers are a lack of knowledge and understanding, as well as a lack of opportunity, inconvenience, and task difficulty [75]; therefore, the government should emphasize the promotion of waste classification methods for the whole of society, and it should urge them to fulfill their social responsibilities. The government can strengthen the environmental awareness of residents through various channels, in accordance with the characteristics of the region. Waste-related activities and information must coalesce with people’s daily activities and concerns, so that households have a clear recycling plan and know what, where, when, and how to recycle. Paying more attention to improving residents’ awareness of what environmental concerns exist, and removing barriers so that residents can participate in waste sorting, may help to improve the participation rate.

### 5.3. Strengths and Limitations

This paper makes the following contributions. First, we adopted a hybrid method, combining NCA and fsQCA, to analyze the complex causalities between external factors and residents’ participation in waste classification; this is conducive to promoting the development of studies concerning the necessary and sufficient relationships between the factors behind the participation rate. Second, although previous studies have discussed the factors influencing residents’ participation in waste sorting, this paper is an empirical study that examines the interaction between external factors influencing the residents of pilot cities from various aspects. It explores four pathways that achieve a high participation rate, thus expanding the research concerning residents’ participation in waste sorting in developing countries. Third, this paper provides a comprehensive insight into the different developmental stages of waste sorting policies, as well as the advantages of these policies; therefore, it can provide inspiration for policy choices in this field. 

There are still some limitations of this paper, which are worthy of further study. First, limited by the availability of data, we only analyzed the pathways concerning the participation rate of some urban residents who first implemented waste classification policies; hence, in future studies, we can obtain more sufficient data via questionnaires and surveys, and thus, further understand the psychological factors behind participation in the waste sorting process, as well as improve the configuration of the pathways. Further analysis of such configurations can be conducted in the future. Moreover, we only discussed planar static data; in future studies, we can further examine the temporal and spatial correlation. In addition, we will also pay attention to the effective changes in residents’ behavior brought about by the policies.

## 6. Conclusions

This study focuses on the problem of resident participation in waste sorting in pilot cities in China. It provides an in-depth analysis of the differences between the pathways in the literature concerning MSW. The analysis of waste classification clarifies the impact of external factors on residents’ participation in the waste sorting process, and it provides an outline of different waste management practices in areas with different resource and development patterns. As China intends to achieve carbon neutrality and establish a circular economy, analyzing participation in waste classification is of great value. Combining NCA and fsQCA methods, this study empirically studied the external factors relating to waste management in Chinese cities by configuring a combination of factors. This paper makes the following conclusions: 

First, the NCA method was adopted, and it found that no single external factor constitutes a necessary condition for a high participation rate; however, improving environmental quality can make a significant contribution. This means that the complex issue of participation in waste sorting needs to be studied from an integrated perspective that takes external factors into account.

Second, fsQCA analysis also proved the asymmetry of causality. We found four pathways that demonstrate how to achieve a high participation rate. These reflect the multiple ways in which a high participation rate can be achieved in different cities. Convenience always tends to have a positive effect on the participation rate. Moreover, environmental quality and economic factors have different effects on different pathways. In terms of core factors, H1 and H2, H3, and H4 share the same core conditions; therefore, they are divided into environment-driven pathways and resource-driven pathways. An environment-driven pathway does not require advocacy policies, whereas a resource-driven pathway requires a combination of advocacy policies and convenience. In addition, whether economic factors and compulsory policies constitute marginal conditions depends on their presence or absence in H3 and H4; however, they remain consistent in their respective pathways.

Third, in the configurations NH1 and NH2, which comprise conditions that contribute to low participation rates, environmental quality appears to be a core variable, accompanied by compulsory and advocacy policies. In particular, for NH3, even if all other variables are present, the lack of convenience directly leads to low participation rates.

## Figures and Tables

**Table 1:** Results of the calibration anchor points.

Sets	Totally Insubordinate	Intersection	Totally Subordinate
Participation rate	90.01	75.63	40.67
Economic factors	498.99	451.27	283.561
Environmental quality	165,388.80	90,534.50	50,590.10
Convenience	8611.40	2502.00	341.20
Mandatory policies	4.80	2.01	0.01
Advocacy policies	6.80	2.01	1.01

**Table 2:** Variables and their descriptive statistics.

Variables	Measurements	Mean Value	Standard Deviation	Maximum Value	Maximum Value
Participation rate	Participation rate regarding municipal solid waste classification.	70.53	15.50	95.50	40
Economic factors	GDP per capita.	105,920.56	42,850.13	203,489	36,546
Mandatory policies	Number of mandatory policies issued by local governments to solve problems relating to waste classification.	1.92	1.35	5	0
Advocacy policies	Number of advocacy policies issued by local governments to solve problems relating to waste classification.	3.12	2.25	10	1
Environmental quality	Weighted linear method was used to calculate environmental value.	429.56	70.46	530.26	259
Convenience	Number of special vehicles used for sanitary purposes.	3571	2807.20	12,526	252

**Table 3:** NCA method results.

Condition	Method	Accuracy	Ceiling Zone	Scope	Effect Size	*p*-Value
Environmental quality	CR	88%	0.19	0.90	0.21	0.05
	CE	100%	0.19	0.90	0.22	0.02
Mandatory policies	CR	92%	0.08	0.86	0.09	0.09
	CE	100%	0.88	0.86	0.10	0.16
Advocacy policies	CR	100%	0.06	0.88	0.07	0.22
	CE	100%	0.12	0.88	0.13	0.18
Economic factors	CR	84%	0.17	0.91	0.19	0.11
	CE	100%	0.06	0.91	0.06	0.48
Convenience	CR	100%	0.02	0.89	0.01	0.74
	CE	100%	0.03	0.89	0.03	0.72

Note: A 0.1 ≤ effect size < 0.3; “Moderate effect”; B 0.3 ≤ level of impact < 0.5; “Advanced effect”.

**Table 4:** Results of the analysis of a combination of necessary conditions performed via bottleneck analysis.

Variables	Environmental Quality	Economic Factors	Convenience	Mandatory Policies	Advocacy Policies
0	NN	NN	NN	NN	NN
10	NN	NN	NN	NN	NN
20	NN	NN	NN	NN	NN
30	NN	NN	NN	NN	NN
40	NN	NN	NN	NN	NN
50	3.70	NN	NN	NN	NN
60	19.10	5.20	NN	4.60	NN
70	34.50	25.90	NN	14.00	NN
80	49.80	46.60	NN	23.40	14.20
90	65.20	67.30	NN	32.80	30.50
100	80.60	88.00	82.10	42.20	46.80

Note: NN means no necessity.

**Table 5:** QCA necessity test.

	Outcome Variable: fzwsp		Outcome Variable: ~fzwsp	
	Consistency	Coverage	Consistency	Coverage
fzeq	0.78	0.72	0.63	0.58
~fzeq	0.55	0.60	0.70	0.76
fzef	0.68	0.63	0.61	0.65
~fzef	0.56	0.60	0.61	0.65
fzcv	0.77	0.77	0.55	0.55
~fzcv	0.55	0.55	0.77	0.77
fzms	0.67	0.77	0.52	0.60
~fzms	0.65	0.58	0.80	0.71
fzap	0.63	0.60	0.63	0.60
~fzap	0.59	0.62	0.59	0.61

Note: fzwsp is the fuzzy set for the participation rate; fzeq is the fuzzy set for environmental quality; fzef is the fuzzy set for economic factors; fzcv is the fuzzy set for convenience; fzms is the fuzzy set for mandatory policies; and fzap is the fuzzy set for advocacy policies. “~” indicates non.

**Table 6:** High and low participation configurations, as per fsQCA analysis.

Conditions	H1	H2	H3	H4	NH1	NH2	NH3
Environmental quality	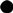	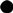		⊗	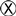	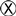	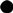
Economic factors	⊗	⊗	•	⊗		⊗	•
Mandatory policies	⊗		•	⊗	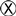	⊗	•
Advocacy policies	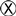	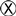	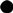	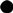		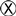	•
Convenience		•	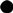	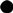	⊗		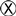
Raw coverage	0.35	0.30	0.47	0.22	0.55	0.36	0.27
Unique coverage	0.09	0.04	0.32	0.04	0.19	0.03	0.11
Consistency	0.85	0.86	0.93	0.87	0.93	0.90	0.88
Solution coverage	0.76				0.69		
Solution consistency	0.88				0.89		

Note: 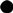 = core conditions exist; 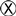 = lack of core conditions; • = edge conditions exist; and ⊗ = missing edge conditions.

**Figure A1:**
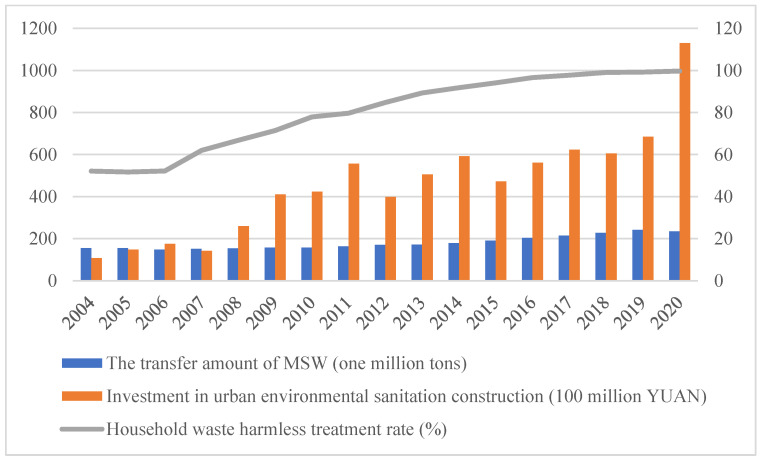
The transfer amount of MSW, investment in urban environmental sanitation construction, and household waste harmless treatment rate. (Data source: the National Bureau of Statistics of China http://www.stats.gov.cn/ (accessed on 11 July 2022)).

**Figure A2:**
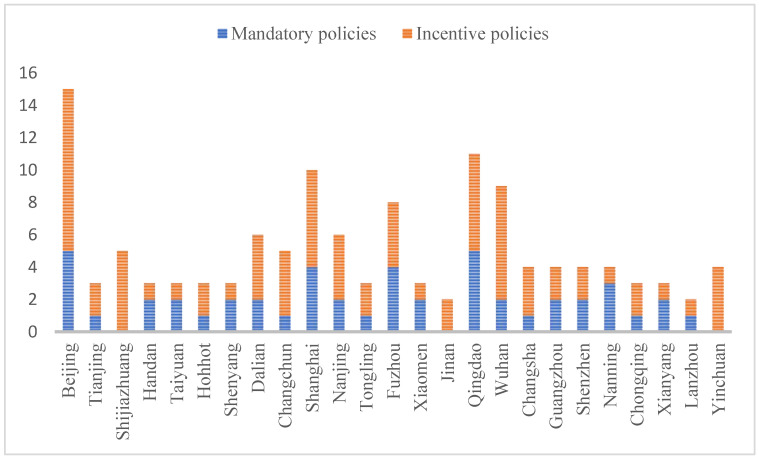
Two types of policy rules concerning MSW sorting.

## Data Availability

The data used to support the findings of this study are available from the corresponding author upon request.

## Supplementary Materials

The following supporting information can be downloaded at: https://www.mdpi.com/article/10.3390/ijerph20054080/s1, Table S1: Literature research results of factors of participation in MSW. References [6,8,9,10,14,15,17,18,20,23,37,40,41,42,44,45,46,48,51,52,53,55,61,73,75,78,79,80,82,83,84,85,86,87,88,89,90,91,92,93,94,95,96,97,98,99,100,101,102,103,104,105,106] are cited in the supplementary materials.

## Appendix A

**Table A1:** The truth table.

fzeq	fzpgdp	fznve	fzms	fzei	number	fzwsp	Raw Consist	PRI Consist	SYM Consist
1	1	1	1	1	1	1	0.954447	0.888888	0.888889
0	1	1	1	1	1	1	0.95443	0.883117	0.883117
1	0	1	1	0	1	1	0.943144	0.738461	0.774193
1	0	0	0	0	1	0	0.90566	0.67742	0.677419
1	0	1	0	0	1	0	0.858667	0.404495	0.404494
0	0	1	0	1	1	0	0.86859	0.359375	0.365079
1	1	0	1	1	1	0	0.69657	0.290123	0.290123
0	0	1	0	0	1	0	0.78836	0.259259	0.259259
0	1	0	0	0	1	0	0.684902	0.152941	0.152941
0	0	0	0	0	1	0	0.672727	0.152941	0.152941
0	1	0	0	1	1	0	0.651852	0.12963	0.12963
0	0	0	0	1	2	0	0.662198	0.0381677	0.0381677
