# Supplementary material for: External Factors Impacting Residents’ Participation in Waste Sorting Using NCA and fsQCA Methods on Pilot Cities in China
Source: Int J Environ Res Public Health. 2023 Feb 24;20(5):4080. doi: 10.3390/ijerph20054080 (PMC10001695; doi:10.3390/ijerph20054080)
Supplement: Supplementary file 1 [file ijerph-20-04080-s001.zip › ijerph-2177226-supplementary.pdf]

TableS1. Literature research results of factors of participation in MSW

Research Area	Internal (Psychological) factors	External factors	References
China	Attitudes, health consciousness, environmental awareness, perceived behavior control, subjective norm	Policies, economic level，population, cultural consumption, knowledge, convenience, educational attainment, institutional trust, social norm, social networks	[1–14]
Changchun, China		Policies, enterprise cooperation	[15]
Shanghai, China	Attitudes, subjective norms, perceived behavioral control	Infrastructure, economic incentives, assistance and supervision, publicity and education, classification standards, classification facilities, the recycling system, knowledge	[16–19]
Jiaxing, China	Attitudes, subjective norms, perceived policy effectiveness		[20]
Fuzhou, China	Perceived value, perceived external pressure	Economic incentives	[21]
Anji, China	Perceived environmental sustainability, perceived individual contribution	Policies	[22]
Chongqing, China	Attitudes	Situational factors, publicity and education	[23]
Chengdu, China	Attitude, perceived behavior control	Knowledge	[24]
Xianmen, China		Social motivation, knowledge, policies	[25]
Hong Kong, China	Perceived policy effectiveness		[26]
Hangzhou, China	Attitudes	Supervision, environment, social-contextual conditions	[27–29]
Zhengzhou, China	Attitudes, peer pressure	Policies, convenience, knowledge	[30]
Jiangsu, China	Environmental awareness, guilt	Publicity and education, convenience, educational attainment, cost	[31–33]
Thailand		Voluntary mechanism, economic incentive mechanism	[34]
Malaysia	Attitudes, subjective norm, perceived behavioral control,		[35]

	moral norm		
Ecuador	Attitudes, social norms, social responsibility	Convenience, economic drivers	[36]
Greek	Attitude, moral norms		[37]
Catalonia, Spain		Geographic, demographic, socioeconomic, policies	[38]
Finland	Attitude, perceived behavioral control	Age, convenience	[39]
Sweden		Waste tariff, convenience, classified information	[40–42]
Ghana	Sorting or health-related perceptions	Economic incentives, gender, income	[43]
Italy	Environmental concern	social capital	[44]
Cebu City, Philippines		Policies, convenience	[45]
Vietnam	Moral norm, attitudes, situational factors, trust in local authority	Policies	[36]
Delhi, India		Knowledge, educational attainment	[47]
Brixworth, UK	Concern of the community	Opportunities, facilities and knowledge, convenience	[48]
England		Culture, economic level, age	[49]
Saudi Arabia	Attitude, social influence, perceived behavioral control	Market incentives, government facilitators	[50]
Literature research	Environmental concern	Education, environmental alteration, convenience, task difficulty, GDP level, separation system	[51–53]

Reference

1. Wu, Z.; Zhang, Y.; Chen, Q.; Wang, H. Attitude of Chinese Public towards Municipal Solid Waste Sorting Policy: A Text Mining Study. *Science of The Total Environment* **2021**, *756*, 142674, doi:10.1016/j.scitotenv.2020.142674.
2. Tian, J.; Gong, Y.; Li, Y.; Chen, X.; Zhang, L.; Sun, Y. Can Policy Implementation Increase Public Waste Sorting Behavior? The Comparison between Regions with and without Waste Sorting Policy Implementation in China. *Journal of Cleaner Production* **2022**, *363*, 132401, doi:10.1016/j.jclepro.2022.132401.
3. Chu, X.; Chu, Z.; Wang, X.; Huang, W.-C.; Ni, Y. Comparative Analysis on the Performances of Implementing Compulsory and Advocative Policies in Municipal Solid Waste Classification. *Environmental Impact Assessment Review* **2023**, *99*, 106982, doi:10.1016/j.eiar.2022.106982.
4. Xu, X.; Wang, S.; Yu, Y. Consumer's Intention to Purchase Green Furniture: Do Health Consciousness and Environmental Awareness Matter? *Science of The Total Environment* **2020**, *704*, 135275, doi:10.1016/j.scitotenv.2019.135275.
5. Wang, A.; Dang, S.; Luo, W.; Ji, K. Cultural Consumption and Knowledge, Attitudes, and

Practices Regarding Waste Separation Management in China. *IJERPH* **2021**, *19*, 338, doi:10.3390/ijerph19010338.
6. Wang, H.; Gui, H.; Ren, C.; Liu, G. Factors Influencing Urban Residents' Intention of Garbage Sorting in China: An Extended TPB by Integrating Expectancy Theory and Norm Activation Model. *Sustainability* **2021**, *13*, 12985, doi:10.3390/su132312985.
7. Yin, H.; Huang, Y.; Wang, K. How Do Environmental Concerns and Governance Performance Affect Public Environmental Participation: A Case Study of Waste Sorting in Urban China. *IJERPH* **2021**, *18*, 9947, doi:10.3390/ijerph18199947.
8. Cheng, X.; Long, R.; Yang, J. Interactive Effects of Two-Way Information and Perceived Convenience on Waste Separation Behavior: Evidence from Residents in Eastern China. *Journal of Cleaner Production* **2022**, *374*, 134032, doi:10.1016/j.jclepro.2022.134032.
9. Shen, J.; Zheng, D.; Zhang, X.; Qu, M. Investigating Rural Domestic Waste Sorting Intentions Based on an Integrative Framework of Planned Behavior Theory and Normative Activation Models: Evidence from Guanzhong Basin, China. *IJERPH* **2020**, *17*, 4887, doi:10.3390/ijerph17134887.
10. Wang, H.; Liu, X.; Wang, N.; Zhang, K.; Wang, F.; Zhang, S.; Wang, R.; Zheng, P.; Matsushita, M. Key Factors Influencing Public Awareness of Household Solid Waste Recycling in Urban Areas of China: A Case Study. *Resources, Conservation and Recycling* **2020**, *158*, 104813, doi:10.1016/j.resconrec.2020.104813.
11. Kuang, Y.; Lin, B. Public Participation and City Sustainability: Evidence from Urban Garbage Classification in China. *Sustainable Cities and Society* **2021**, *67*, 102741, doi:10.1016/j.scs.2021.102741.
12. Wang, Y.; Hao, F. Public Perception Matters: Individual Waste Sorting in Chinese Communities. *Resources, Conservation and Recycling* **2020**, *159*, 104860, doi:10.1016/j.resconrec.2020.104860.
13. Luo, H.; Zhao, L.; Zhang, Z. The Impacts of Social Interaction-Based Factors on Household Waste-Related Behaviors. *Waste Management* **2020**, *118*, 270–280, doi:10.1016/j.wasman.2020.08.046.
14. He, C.; Fu, Y. Why Does Waste Separation Policy Vary across Different Chinese Cities? A Configurational Analysis of the Pilot Scheme. *Journal of Cleaner Production* **2021**, *283*, 124613, doi:10.1016/j.jclepro.2020.124613.
15. Steuer, B.; Li, H. An Effective System for Recovering Recyclable Waste from Households in China: Ant Recovery's Bottom-up Scheme in Changchun City. *Waste Management* **2022**, *139*, 352–361, doi:10.1016/j.wasman.2021.12.039.
16. Govindan, K.; Zhuang, Y.; Chen, G. Analysis of Factors Influencing Residents' Waste Sorting Behavior: A Case Study of Shanghai. *Journal of Cleaner Production* **2022**, *349*, 131126, doi:10.1016/j.jclepro.2022.131126.
17. Tchounwou, P. Influencing Factors on the Household-Waste-Classification Behavior of Urban Residents: A Case Study in Shanghai. *IJERPH* **2022**, *1*, 1–2, doi:10.3390/ijerph2004010001.
18. Wang, H.; Li, J.; Mangmeechai, A.; Su, J. Linking Perceived Policy Effectiveness and Proenvironmental Behavior: The Influence of Attitude, Implementation Intention, and Knowledge. *IJERPH* **2021**, *18*, 2910, doi:10.3390/ijerph18062910.
19. Wang, X.; Tzeng, S.-Y.; Mardani, A. Spatial Differentiation and Driving Mechanisms of Urban Household Waste Separation Behavior in Shanghai, China. *Technological Forecasting and*

Social Change **2022**, *181*, 121753, doi:10.1016/j.techfore.2022.121753.
20. Liu, Q.; Xu, Q.; Shen, X.; Chen, B.; Esfahani, S.S. The Mechanism of Household Waste Sorting Behaviour—A Study of Jiaxing, China. *IJERPH* **2022**, *19*, 2447, doi:10.3390/ijerph19042447.
21. Zhang, L.-P.; Zhu, Z.-P. Can Smart Waste Bins Solve the Dilemmaof Household Solid Waste Sorting in China?A Case Study of Fuzhou City. *Pol. J. Environ. Stud.* **2020**, *29*, 3943–3954, doi:10.15244/pjoes/115868.
22. Ling, M.; Xu, L. Incentivizing Household Recycling Crowds out Public Support for Other Waste Management Policies: A Long-Term Quasi-Experimental Study. *Journal of Environmental Management* **2021**, *299*, 113675, doi:10.1016/j.jenvman.2021.113675.
23. Yang, X.; Chen, X.; Xiao, X.; Xi, H.; Liu, S. College Students' Willingness to Separate Municipal Waste and Its Influencing Factors: A Case Study in Chongqing, China. *Sustainability* **2021**, *13*, 12914, doi:10.3390/su132212914.
24. Zhang, S.; Hu, D.; Lin, T.; Li, W.; Zhao, R.; Yang, H.; Pei, Y.; Jiang, L. Determinants Affecting Residents' Waste Classification Intention and Behavior: A Study Based on TPB and A-B-C Methodology. *Journal of Environmental Management* **2021**, *290*, 112591, doi:10.1016/j.jenvman.2021.112591.
25. Xiao, L.; Zhang, G.; Zhu, Y.; Lin, T. Promoting Public Participation in Household Waste Management: A Survey Based Method and Case Study in Xiamen City, China. *Journal of Cleaner Production* **2017**, *144*, 313–322, doi:10.1016/j.jclepro.2017.01.022.
26. Wan, C.; Shen, G.Q.; Yu, A. The Moderating Effect of Perceived Policy Effectiveness on Recycling Intention. *Journal of Environmental Psychology* **2014**, *37*, 55–60, doi:10.1016/j.jenvp.2013.11.006.
27. Zheng, B.; Wan, S.; Wen, J.; Ye, L.; Lv, K. Do Public Awareness and Behaviors in Rural Domestic Waste Classification Help Reduce COVID-19? A Case Study in China. *Pol. J. Environ. Stud.* **2021**, *30*, 3897–3906, doi:10.15244/pjoes/131008.
28. Ling, M.; Xu, L.; Xiang, L. Social-Contextual Influences on Public Participation in Incentive Programs of Household Waste Separation. *Journal of Environmental Management* **2021**, *281*, 111914, doi:10.1016/j.jenvman.2020.111914.
29. Xu, L.; Ling, M.; Lu, Y.; Shen, M. Understanding Household Waste Separation Behaviour: Testing the Roles of Moral, Past Experience, and Perceived Policy Effectiveness within the Theory of Planned Behaviour. *SUSTAINABILITY* **2017**, *9*, doi:10.3390/su9040625.
30. Hao, M.; Zhang, D.; Morse, S. Waste Separation Behaviour of College Students under a Mandatory Policy in China: A Case Study of Zhengzhou City. *IJERPH* **2020**, *17*, 8190, doi:10.3390/ijerph17218190.
31. Meng, X.; Tan, X.; Wang, Y.; Wen, Z.; Tao, Y.; Qian, Y. Investigation on Decision-Making Mechanism of Residents' Household Solid Waste Classification and Recycling Behaviors. *Resources, Conservation and Recycling* **2019**, *140*, 224–234, doi:10.1016/j.resconrec.2018.09.021.
32. Cudjoe, D.; Nketiah, E.; Obuobi, B.; Adjei, M.; Zhu, B.; Adu-Gyamfi, G. Predicting Waste Sorting Intention of Residents of Jiangsu Province, China. *Journal of Cleaner Production* **2022**, *366*, 132838, doi:10.1016/j.jclepro.2022.132838.
33. Liu, A.; Osewe, M.; Wang, H.; Xiong, H. Rural Residents' Awareness of Environmental Protection and Waste Classification Behavior in Jiangsu, China: An Empirical Analysis. *IJERPH* **2020**, *17*, 8928, doi:10.3390/ijerph17238928.

34. Boonrod, K.; Towprayoon, S.; Bonnet, S.; Tripetchkul, S. Enhancing Organic Waste Separation at the Source Behavior: A Case Study of the Application of Motivation Mechanisms in Communities in Thailand. *Resources, Conservation and Recycling* **2015**, *95*, 77–90, doi:10.1016/j.resconrec.2014.12.002.
35. Razali, F.; Daud, D.; Weng-Wai, C.; Anthony Jiram, W.R. Waste Separation at Source Behaviour among Malaysian Households: The Theory of Planned Behaviour with Moral Norm. *Journal of Cleaner Production* **2020**, *271*, 122025, doi:10.1016/j.jclepro.2020.122025.
36. Negash, Y.T.; Sarmiento, L.S.C.; Tseng, M.-L.; Lim, M.K.; Ali, M.H. Engagement Factors for Household Waste Sorting in Ecuador: Improving Perceived Convenience and Environmental Attitudes Enhances Waste Sorting Capacity. *Resources, Conservation and Recycling* **2021**, *175*, 105893, doi:10.1016/j.resconrec.2021.105893.
37. Botetzagias, I.; Dima, A.-F.; Malesios, C. Extending the Theory of Planned Behavior in the Context of Recycling: The Role of Moral Norms and of Demographic Predictors. *Resources, Conservation and Recycling* **2015**, *95*, 58–67, doi:10.1016/j.resconrec.2014.12.004.
38. Saldivia-Gonzatti, L.I.; Jannes, G.; Barreal, J. Factors Influencing the Rate of Sorted Solid Waste Collection: An Empirical Analysis towards Local Management in Catalonia (NE Spain). *Cities* **2022**, *131*, 104038, doi:10.1016/j.cities.2022.104038.
39. Reijonen, H.; Bellman, S.; Murphy, J.; Kokkonen, H. Factors Related to Recycling Plastic Packaging in Finland's New Waste Management Scheme. *Waste Management* **2021**, *131*, 88–97, doi:10.1016/j.wasman.2021.05.034.
40. Andersson, C.; Stage, J. Direct and Indirect Effects of Waste Management Policies on Household Waste Behaviour: The Case of Sweden. *Waste Management* **2018**, *76*, 19–27, doi:10.1016/j.wasman.2018.03.038.
41. Bernstad, A. Household Food Waste Separation Behavior and the Importance of Convenience. *Waste Management* **2014**, *34*, 1317–1323, doi:10.1016/j.wasman.2014.03.013.
42. Rousta, K.; Bolton, K.; Lundin, M.; Dahlén, L. Quantitative Assessment of Distance to Collection Point and Improved Sorting Information on Source Separation of Household Waste. *Waste Management* **2015**, *40*, 22–30, doi:10.1016/j.wasman.2015.03.005.
43. Owusu, V.; Adjei-Addo, E.; Sundberg, C. Do Economic Incentives Affect Attitudes to Solid Waste Source Separation? Evidence from Ghana. *Resources, Conservation and Recycling* **2013**, *78*, 115–123, doi:10.1016/j.resconrec.2013.07.002.
44. Aprile, M.C.; Fiorillo, D. Intrinsic Incentives in Household Waste Recycling: The Case of Italy in the Year 1998. *Journal of Cleaner Production* **2019**, *227*, 98–110, doi:10.1016/j.jclepro.2019.04.184.
45. Camarillo, M.E.C.; Bellotindos, L.M. A Study of Policy Implementation and Community Participation in the Municipal Solid Waste Management in the Philippines. *App. Envi. Res.* **2021**, 30–45, doi:10.35762/AER.2021.43.2.3.
46. Loan, L.T.T.; Nomura, H.; Takahashi, Y.; Yabe, M. Psychological Driving Forces behind Households' Behaviors toward Municipal Organic Waste Separation at Source in Vietnam: A Structural Equation Modeling Approach. *J Mater Cycles Waste Manag* **2017**, *19*, 1052–1060, doi:10.1007/s10163-017-0587-3.
47. Bhawal Mukherji, S.; Sekiyama, M.; Mino, T.; Chaturvedi, B. Resident Knowledge and Willingness to Engage in Waste Management in Delhi, India. *Sustainability* **2016**, *8*, 1065, doi:10.3390/su8101065.

48. Tonglet, M.; Phillips, P.S.; Read, A.D. Using the Theory of Planned Behaviour to Investigate the Determinants of Recycling Behaviour: A Case Study from Brixworth, UK. *Resources, Conservation and Recycling* **2004**, *41*, 191–214, doi:10.1016/j.resconrec.2003.11.001.
49. Martin, M.; Williams, I.D.; Clark, M. Social, Cultural and Structural Influences on Household Waste Recycling: A Case Study. *Resources, Conservation and Recycling* **2006**, *48*, 357–395, doi:10.1016/j.resconrec.2005.09.005.
50. Labib, O.A.; Manaf, L.; Sharaai, A.H.; Zaid, S.S.M. Understanding the Effect of Internal and External Factors on Households' Willingness to Sort Waste in Dammam City, Saudi Arabia. *IJERPH* **2021**, *18*, 9685, doi:10.3390/ijerph18189685.
51. Xia, Z.; Gu, Y.; Li, J.; Xie, J.; Liu, F.; Wen, X.; Tian, X.; Zhang, C. Do Behavioural Interventions Enhance Waste Recycling Practices? Evidence from an Extended Meta-Analysis. *Journal of Cleaner Production* **2022**, 135695, doi:10.1016/j.jclepro.2022.135695.
52. Fogt Jacobsen, L.; Pedersen, S.; Thøgersen, J. Drivers of and Barriers to Consumers' Plastic Packaging Waste Avoidance and Recycling – A Systematic Literature Review. *Waste Management* **2022**, *141*, 63–78, doi:10.1016/j.wasman.2022.01.021.
53. Wan, M.; Wan, L. Exploring the Pathways to Participation in Household Waste Sorting in Different National Contexts: A Fuzzy-Set QCA Approach. *IEEE Access* **2020**, *8*, 179373–179388, doi:10.1109/ACCESS.2020.3027978.
